# Melanoma Marjolin’s ulcer in the hand: A case report

**DOI:** 10.1016/j.ijscr.2019.06.029

**Published:** 2019-06-20

**Authors:** Rafaela Pais Serras, David Carvalho Rasteiro, Maria Manuel Mendes, Maria Manuel Mouzinho

**Affiliations:** Department of Plastic and Reconstructive Surgery, Hospital de São José, 1150-199, Lisbon, Portugal

**Keywords:** Marjolin’s ulcer, Melanoma, Burn scar, Skin cancer, Chronic wound, Case report

## Abstract

**Introduction:**

Burn scars have the potential to malignant transformation, creating an identity named Marjolin’s ulcer. This term refers to any scar in a traumatized skin that degenerates into a skin cancer. The majority of them are derived from burns that were not grafted, appearing several years later. The most frequent type is squamous cell carcinoma with few cases of melanoma described in the literature. This last one accounts for a poor prognosis.

**Case presentation:**

Female, 74 years old, presents to our department with a malignant melanoma arising from a thermal burn scar in the right thenar eminence 14 years after the initial injury. The lesion was excised and the defect covered by a full thickness skin graft. At 6 months post-operatively there are no signs of locoregional recurrence or systemic dissemination.

**Discussion:**

Malignant melanoma arising from a burn scar is extremely rare and the pathogenesis is unknown. Many theories try to explain this low incidence and the factors that contribute to degenerative changes of melanocytes in the affected skin.

**Conclusion:**

Health professionals must consider this pathology when evaluating a burn scar or a chronic wound, performing a biopsy when suspicion is high. An early diagnosis, a prompt surgical intervention and a greater vigilance are the keys to success and survival.

## Highlights

• Marjolin’s ulcer is usually misdiagnosed.
• Some theories try to explain the mechanisms of malignant degeneration.
• Melanoma is rare and aggressive and, once suspected, a biopsy must be done.
• Deep second and third degree burns must be debrided and covered by a skin graft.
• Early diagnosis, treatment and vigilance are the keys to success and survival.

## 1 Introduction

Marjolin’s ulcer is a rare and aggressive tumor that arise in scar tissue, particularly in burn scars. It was described for the first time in 1828 by Jean-Nicolas Marjolin. Since then, some cases have been reported in the literature.

This term refers to any malignant transformation of a previously traumatized skin, but the squamous cell carcinoma is the most frequent histological type, followed by basal cell carcinoma and rarely melanoma or sarcoma.

Deep partial thickness and full thickness burns healed by secondary intention, without grafting, are more prone to develop this type of cancer. The average time of latency has been described in the literature as 23–37 years [1].

There is no distribution for race or age, but it occurs mostly in fifth decade of life [2], with a higher preponderance in men. It can occur in all anatomic locations, but mostly in lower extremities (>40%), followed by upper extremities, head, neck and trunk [3].

Once, it has high recurrence and metastatic rates, it is important a diagnostic and treatment without delay.

A case of melanoma Marjolin’s ulcer arising from a burn scar located in thenar eminence will be described. This type of cancer in traumatized skin and this location is not common and there are only a few cases described in the literature. A brief review of this thematic will be done.

The present work has been reported in line with the SCARE criteria [4].

## 2 Case presentation

The patient is a 74 years old, skin phototype III of the Fitzpatrick scale, right-hand-dominant female, who suffered a deep partial thickness burn with hot water in the right hand 14 years prior to admission. This burn healed by secondary intention. No debridement or skin graft was performed. There was no family history of skin cancer.

**Fig. 1:**
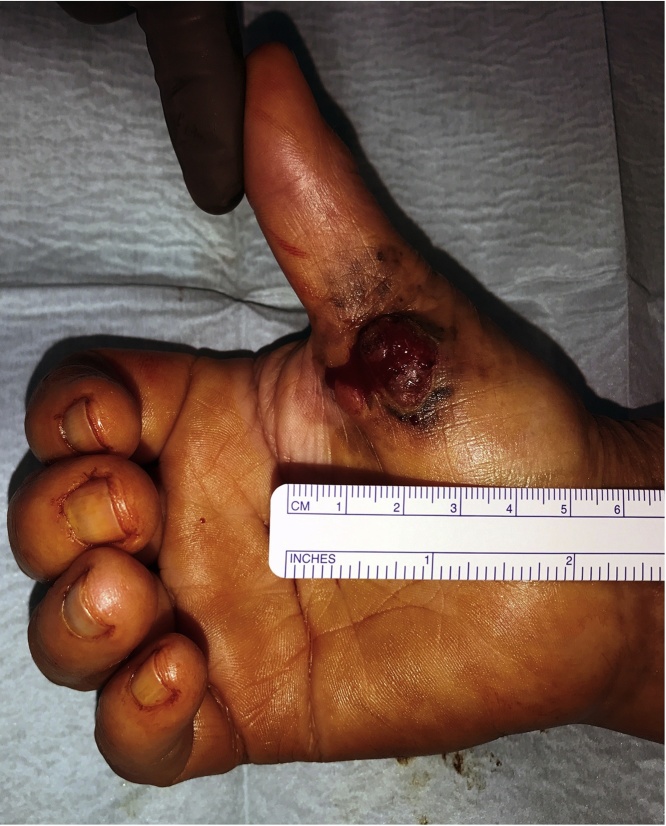
Preoperative view: presence of tumor in thenar eminence.

She presented to our department in January 2018 with an exophytic lesion in the right thenar eminence, about 2 cm of diameter, painful and bleeding, that had increased in size during the past several years (Fig. 1).

Her neurovascular exam was intact. She had no palpable axillary lymphadenopathy.

Laboratorial analyses and ultrasound revealed no relevant changes.

**Fig. 2:**
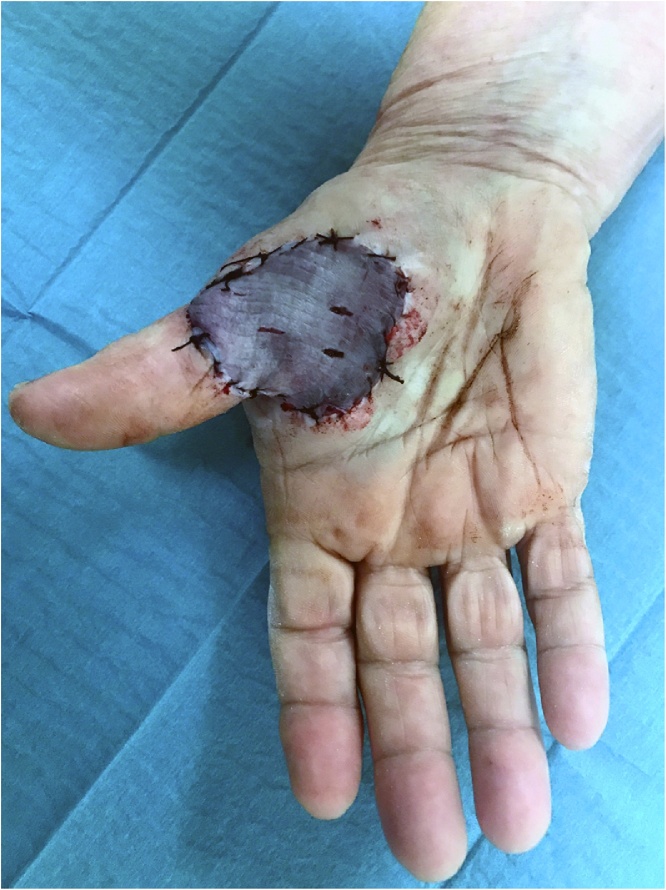
Intraoperative view: reconstruction of defect with a full thickness skin graft.

The treatment was local excision of the lesion with a margin of 1 cm of healthy tissue and then the defect was covered by a full thickness skin graft harvested from the internal surface of the right arm (Fig. 2), under general anesthesia.

**Fig. 3:**
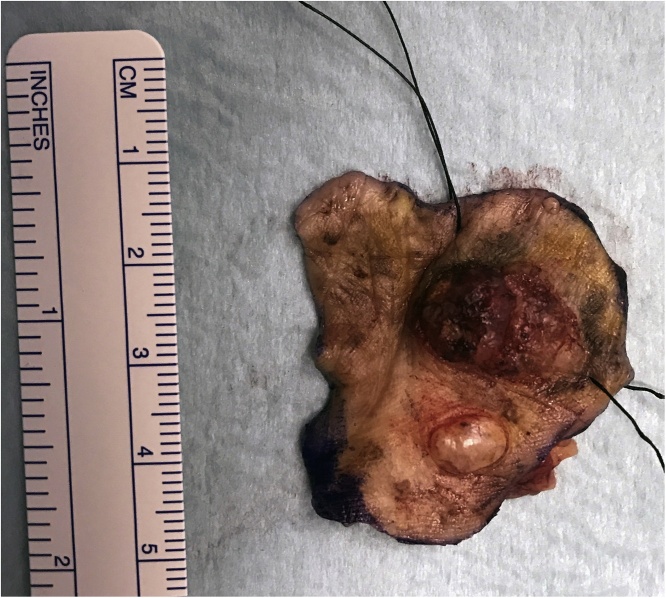
Local excision of the lesion: Macroscopic view of the tumor.

The lesion was sent to microscopic examination (Fig. 3).

No lymph node dissections was carried out at that time.

The anatomophatological examination of excised tissue showed two nodular lesions with focal ulceration compatible with the diagnosis of a superficial spreading melanoma, IV level of Clark, 3,4 mm of the Breslow’s tumor thickness classification with high mitotic rate (13/mm^2^) and linfovascular invasion. pTNM: pT3bNXMX.

A thoracic-abdominal-pelvic computed tomography scan showed no evidence of metastatic disease or axillary lymph node involvement.

The patient was transferred to an oncologic institution where an enlargement of the margins and a sentinel lymph node biopsy was performed. This last one revealed intranodal metastases, so an axillary ganglionar emptying was performed.

In this case there are some negative prognostic factors, such as a chronic latency, presence of ulceration, high mitotic rate and linfovascular invasion. However, at 6 months post-operatively there are no signs of local recurrence or systemic dissemination. She must maintain follow-up for at least 5 years.

## 3 Discussion

Malignant melanoma in burn scars is an uncommon event. The first mention in the literature was in 1965 by Giblin et al. [5]. Since then, few other cases have been reported.

Within burn scar degeneration, malignant melanoma has a worse prognosis compared with squamous cell carcinoma. The low incidence of this type of skin cancer in burn scars can be partially explained by the fact that there is a decreased number of melanocytes in this type of tissue. The reason why transformation of melanocytes in affected skin occurs is unknown. Although there are some theories, such as Virchow´s theory of chronic irritation that holds that repetitive trauma in this undernourished area acts as a promoter of degenerative changes. Scars are susceptible for trauma because they are elevated, there is lack of collagen organization and vascular supply is compromised due to fibrosis that obliterates the vessels [5]. These factors contribute to weaken the neoepithelium. Other factors have been associated like genetic mutations in Fas, p53 e HLA DL4, UV radiation, production of carcinogenic toxins by the scar tissue and impairment of immunological activity due to obliteration of lymphatic vessels by fibrosis [6].

Easy exposure and repetitive trauma in extremities that activate inflammatory mechanisms can explain partially why this is the main anatomic location.

Not all burn scars undergo malignant degeneration, but 0,77–2,0% of them do [7]. It should be suspected according to patient’s history and clinical signs of malignancy, for example, appearance of an exophytic lesion or ulceration, painful, bleeding, with drainage of purulence and unhealing for several months of conservative treatment. In these circumstances a biopsy must be performed to exclude a malignant transformation.

The diagnosis is based on anamnesis, clinical findings and histological examination. An early diagnosis avoids an extensive surgical excision with greater morbidity and mortality.

The treatment depends on TNM classification, differentiation grade, existence of viable tissue surrounding the lesion, age and patient comorbidities. The gold standard is excision with 2–4 cm safety margins and reconstruction of the defect with skin graft or local or free flap. Chemotherapy or radiotherapy adjuvant or neoadjuvant can be associated and proximal amputation can be necessary if there is involvement of the bone or extensive destruction of the structures.

No consensus was obtained regarding the time of follow-up, but is universally accepted that this group of patients must be under a rigid vigilance to reduce morbidity and mortality.

Further investigation is necessary to understand the exact mechanism that leads to malignant degeneration in burn scars, in many cases several years after initial injury. The time of follow-up must be determined to reduce the chances of recurrence and systemic dissemination.

Prevention is the best approach, so deep partial thickness burns and full thickness burns must be treated surgically by excision and skin graft.

## 4 Conclusion

Health professionals must consider this pathology when evaluating a burn scar or a chronic wound, performing a biopsy when suspicion is high. Unfortunately, the diagnosis and treatment are usually delayed, which accounts for poor prognosis.

An early diagnosis, a prompt surgical intervention and a greater vigilance are the keys to success and survival.

## Conflicts of interest

There are no conflicts of interest.

## Sources of funding

There are no sources of funding in this paper.

## Ethical approval

Ethical approval for the submission of this case report has been exempted by our institution.

## Consent

Written informed consent was obtained from the patient for publication of this case report and accompanying images.

## Author’s contribution

Rafaela Pais Serras – Third surgeon of the case, data collection, data analysis, writing the paper.

David Carvalho Rasteiro – First surgeon of the case, data analysis.

Maria Manuel Mendes – Second surgeon of the case, data analysis.

Maria Manuel Mouzinho – Article supervision.

## Registration of research studies

Not applicable.

## Guarantor

Rafaela Pais Serras.

## Provenance and peer review

Not commissioned, externally peer-reviewed.
